# The effects of academic adaptability on academic burnout, immersion in learning, and academic performance among Chinese medical students: a cross-sectional study

**DOI:** 10.1186/s12909-019-1640-9

**Published:** 2019-06-13

**Authors:** Yu Jin Xie, De Pin Cao, Tao Sun, Li Bin Yang

**Affiliations:** 1 School of Public Health, Harbin Medical University, Harbin, China; 2 Center for Higher Education Research and Teaching Quality Evaluation, Harbin Medical University, Harbin, China

**Keywords:** Academic adaptability, Academic burnout, Immersion in learning, Academic performance, Chinese medical students

## Abstract

**Background:**

Medical students struggle with a heavy workload during their comparatively long course of study in China. The future of medical science depends largely on whether or not medical students become qualified. This study aims to explore whether medical students’ academic adaptability affects learning outcomes. This paper will not only provide scientific evidence for educators and administrators of medical schools but will also benefit students by improving their aptitude and adaptability through a thorough discussion on their educational environment.

**Methods:**

We conducted a cross-sectional survey from September to December 2016. A total of 1977 respondents completed the questionnaire with a response rate of 79.08%. A cross-sectional survey was used in this study. Descriptive statistics, factor analysis, General Linear Model (GLM) analysis, standard multiple regression, and hierarchical multiple regression were performed for data analysis using the Statistical Package for Social Sciences software (SPSS Version 19.0, SPSS Inc., Chicago, IL, USA).

**Results:**

Out of the 1977 students, 1586 (80.2%) had mean academic adaptability levels over 3. Findings suggested that academic adaptability (Mean = 3.32), immersion in learning (Mean = 3.20), and academic performance (Mean = 3.39), were at the middle level while academic burnout (Mean = 2.17) was at a low level. Academic adaptability of medical students showed a significant negative relation to academic burnout (Beta = − 0.705, *P*<0.01), there was a significant positive relation between academic adaptability and immersion in learning (Beta = 0.655, *P*<0.01) and academic performance (Beta = 0.407, *P*<0.01).

**Conclusions:**

Higher levels of academic adaptability are associated with lower levels of burnout and higher levels of immersion in learning and academic performance. It might be helpful for medical schools to consider academic adaptability and ways of enhancing such skills in order to enhance student performance and engagement while in school.

## Background

In today’s world, knowledge is constantly being updated. ‘Learning to learn’ and ‘lifelong learning’ have become the general consensus of society. However, the study of medicine is more complicated, which highlights professional, autonomous, and exploratory characteristics. The importance of learning has been widely and deeply recognized, and educators, psychologists and researchers have attached increasing importance to academic adaptability. Since the 1980s, researchers [1] have conducted a lot of theoretical and practical research on the academic adaptability of college students, and have accomplished a series of research achievements, which are gradually becoming extensive. Regarding the concept of academic adaptability, Chinese scholars mostly cite the definition in the *Handbook of Academic Adaptability Test* edited by Professor Chou Bucheng, a famous Chinese psychologist. This definition states that academic adaptability refers to the tendency of individuals to overcome difficulties and achieve better academic effects [2].

College is a vital transition period for student development and adaptability is an important factor for success success in college. Medical students are in a critical period of physical and mental development while they are acquiring knowledge and skills. Furthermore, in college, students develop into independent adults with responsibilities in society. Since most medical students will inevitably become doctors and specialize in an exceptional occupation, they experience more psychological pressure [3] and academic rigor compared to other students in college. Whether medical students can academically adapt to the requirements of the environment and stimulate their greatest learning potential will be related to directly determine their learning effect and even their career development prospects. If this problem is not solved, it will have great adverse effects on social development.

From the early 1980s, researchers [1] have focused on college students’ academic adaptability with long term studies and have developed many authoritative scales. Research on academic adaptability and how to measure it originated from Europe, which is the source of most studies on college students’ academic adaptability. Relevant research focused mainly on the factors that affect academic adaptability and its measurement. For example, Credé and Niehorster [4], in a meta-analysis of 237 studies, found that students’ search for educational resources and their attitude towards learning influence their academic adaptability during their studies. Students with lower degrees of adaptability face many obstacles. To explore factors that influence adaptability, Boulter studied the relationship between self-concept and how freshmen adjusted to school. The results showed that freshmen’s self-awareness and support from teachers could affect students’ academic adaptability [5]. Amy Strage and Gregory P. Hickman have identified a relationship between parenting styles and students’ adaptability through relevant research. Academic adaptability plays a hugely positive role [6]. Frederick, T. et al [7] think that coping styles can effectively predict the adaptability of college students. According to them, a positive coping style is a predictive factor of academic adaptability. Hilary Gerdes’ follow-up survey of college students’ expectations and academic adaptation shows that the differences in students’ emotional and social adaptation are greater than those in students’ learning adaptation [8].

The most widely-recognized measuring tool of college students’ adaptability is the Student Adaptation to College Questionnaire (SACQ) compiled by Baker and Siryk [9]. The questionnaire evaluates many facets of adaptability, including academic adaptability, social adaptability, and emotional adaptability. This scale has a strong reputation for validity and is used internationally. However, these questionnaires do not focus solely on academic adaptability. Simon L. and Roland R. specifically aimed at researching the academic adaptability of freshmen and therefore developed the college student response and adaptability questionnaire (TRAC) [10]. This questionnaire contains 50 items. Academic adaptability is viewed from the perspective of the college student’s personality and is divided into faith, emotion, and behaviour. Behaviour includes nine factors: fear of failure, test anxiety, exam preparation, attention to quality, help received, help given to teachers, learning to learn, effective learning methods, and ease of evaluating college students’ academic adaptability.

The current research in China summarizes the concept, composition, and progress of academic adaptability, and provides guidance and support for us to further the research on students’ academic adaptability. However, there is a lack of systematic research on the current situation, and on results caused by medical students’ academic adaptability. Therefore, the systematic research on medical students’ academic adaptability is still in the exploratory stage. The present study investigates academic adaptability among medical students. We used forward-looking method for raising some hypothesises and randomly interviewed 260 students at the campus of Harbin Medical University. According to the interview results, we explanatorily asserted that academic adaptability is potentially related to academic burnout, immersion in learning, and academic achievement.

The concept of “burnout” was first proposed in 1974. This concept was gradually accepted as a work-related trouble. A series of research studies identified burnout as an obstacle among college students in this particular young group [11]. In 1981, Pines and Katry carried out a comparative study aimed at helping college students and professional workers. They found that the degree of burnout among college students was greater than that among professional workers at work, and they first proposed the concept of academic burnout [12]. This study suggests that students’ academic burnout is the result of long-term academic pressure or load and energy consumption, the gradual decrease in students’ enthusiasm due to school work and activities, indifference and alienation, a phenomenon and the result is not as expected and the negative attitude of schoolwork. This definition, and later research studies on academic burnout, basically use Maslach’s concept of burnout, but only slightly adjust its background.

Following a study on scientists, writers, and athletes in the 1970s, American psychologist Csikszentmihalyi found that when people are engaged in a controllable and challenging activity that requires skills, and is motivation driven, they experience a unique state of ecstasy and a kind of ‘flow’ known as ‘flow experience’ [13]. Flow experience is one of the core concepts of positive psychology. To sum up, the definition of immersion in the learning experience in this study is the deep psychological state of clear goals, comfortable and inner pleasure and the consequent balance between individual ability and the challenge of learning tasks resulting in a positive emotional experience when students are fully engaged in learning activities.

The present study investigates academic adaptability among medical students. We wanted to describe the association if any between academic adaptability, academic burnout, immersion in learning and academic performance. The study will not only provide scientific guidance for educators and administrators of medical schools but will also improve the students’ familiarity with the nature of academic adaptability in order to guide their medical education.

## Methods

### Research design and sample

A cross-sectional survey was used in this study. We began the study in September 2016 and recruited 2500 medical students from four schools. These students attended Harbin Medical University, Qiqihar Medical University, Jiamusi University, and Chengdu Medical College. Subjects were selected using stratified-cluster random sampling and were stratified by levels. This study adopts the online survey and anonymous filling method, and does not involve privacy and sensitive topics. The questionnaire survey was conducted through the online survey platform called ‘questionnaire star’, and the contact person sent direct links to students who voluntarily filled in the questionnaire. The students were promised that the results were only used to write papers and that no single questionnaire analysis was conducted. Lucky rewards were offered in the questionnaire system to encourage students to participate in the study and to ensure the quality of the questionnaire. This investigation is in line with the relevant requirements of the ethics committee. A total of 1977 students returned usable questionnaires, yielding a 79.08% response rate. The returned questionnaires that had obvious errors, incorrect answers on lie-detection questions, or were incomplete were not included in the final sample.

### Measuring instruments

#### Academic adaptability

Academic adaptability was measured using the Chinese version of the University Academic Adaptability Questionnaire (UAAQ) [14]. The original scale, Cronbach’s alpha, was 0.890. The scale showed good reliability and construct validity. The initial scale consisted of 55 items. Lie detection questions, leading questions, and low factor loading items were deleted in the initial scale. The first edition of the academic adaptability scale was revised to reflect the characteristics of medical students. Finally, a total of 27 measurement items were selected for the Medical Students’ Academic Adaptability (MSAA) scale, including 16 positive scoring questions and 11 reverse scoring questions. A five-point Likert scale was used, where 1 point represented complete non-conformity and 5 points represented complete compliance. Positive scoring was assigned to positively worded questions, and reverse scoring to questions stated in a negative fashion. The higher the score, the higher the level of academic adaptability. The Cronbach’s α coefficient of the scale in this study was 0.884.

#### Academic burnout

Academic burnout was measured using the Chinese version of the Maslach Burnout Inventory - Educators Survey (MBI-ES), developed by Schaufeli et al [15]*.* MBI (Maslach Burnout Inventory) is the most widely used burnout measurement tool in the world. In the published empirical research on job burnout, more than 90% of papers and research reports use MBI scale as a measurement tool. MBI has been widely used and tested since it was launched. It has been proved to have good internal consistency reliability, retest reliability, structural validity, conceptual validity, and discriminant validity among others [16, 17]. The MBI-SS consists of 15 items that constitute three scales: emotional exhaustion (five items), depersonalization (four items), and personal accomplishment (six items). All items are scored on a seven-point frequency rating scale ranging from 0 (never) to 6 (always). The Cronbach’s α coefficient of the scale in this study was 0.903.

#### Immersion in learning

Immersion in learning was measured using the Chinese version of Bakker’s Work-Related Flow Inventory (WOLF) [18]. Currently, WOLF is mainly used in the field of organizational behaviour. After modification to make the scale more suitable for an educational context, it contains absorption (four items), enjoyment (four items), and intrinsic learning motivation (five items). These three dimensions contain 13 items in total. Each item is scored on a five-point Likert scale, ranging from 1 (never) to 5 (always). Absorption refers to a state of high concentration, a preoccupation with learning, and a student’s disregard of his or her surroundings. Students who enjoy learning and feel happy in school have a very positive opinion about the quality of their study life. This attitude is consistent with the flow experience. Intrinsic learning motivation refers to an internal experience of pleasure and satisfaction in learning. It drives the individual to continue to be interested in learning and to devote themselves to it. The Cronbach’s α coefficient of the scale in this study was 0.940.

#### Academic performance

Due to variations in medical students’ professional and curricular situations, first-year students evaluate their academic performance according to the entrance grades in their major, while other grades evaluate their academic performance according to the final exam grades in their major. They used the five-point Likert scale to rate their grades from 1 (worst) to 5 (best).

#### Statistical analysis

In this paper, ‘Questionnaire Star’ was used to directly generate the data in SAV format, and SPSS 19.0 software was used for statistical analysis of the data. Descriptive statistical analysis was used to analyse the general situation of the survey subjects of academic adaptability, the current level of academic adaptability and the mean and standard deviation of each factor. The structural validity of the adaptability scale was tested using exploratory factor analysis. Cronbach’s alpha coefficient test method was used to test the reliability of the questionnaire. Kaiser-Meyer-Olkin and Bartlett’s tests were employed to ensure the completion of factor analysis (KMO = 0.929 > 0.9, *P* < 0.001). Pearson correlation analysis was used to examine the correlation between academic adaptability and academic burnout, immersion in learning, and academic performance of medical students. The effect of the academic adaptability of medical students and their internal relations were explored using multiple linear regression analysis. *P* < 0.05 and *P* < 0.01 were statistically significant.

## Results

### Socio-demographic information of participants

Of the 2500 questionnaires sent out, 1977 (79.08%) were considered valid (with approximate 80% of questions in the questionnaire answered) and suitable for analysis. Some general background data on the medical students’ demographic information are shown in Table 1.

**Table 1:** Summary statistics for key variables (*N* = 1977)

	Participants (*N*)	Mean ± SE or % of total *N*
Demographic information		
Gender		
Male	570	28.8%
Female	1407	71.2%
Age	1977	19.90 ± 1.67
Residence		
Urban	1140	57.7%
Rural	837	42.3%
Grade		
One	948	48.0%
Two	383	19.4%
Three	301	15.2%
Four	214	10.8%
Five	131	6.6%
Student type		
Student leaders	1048	53.0%
Ordinary students	929	47.0%

### Reliability and validity analysis

The internal consistency reliability for the 27-item MSAA had an overall Cronbach’s α coefficient of 0.884. Internal consistency reliability analyses showed good internal consistency for the MSAA. The six dimensions of Cronbach’s alpha were 0.867, 0.846, 0.794, 0.770, 0.779, and 0.768, respectively. The Bartlett spherical test coefficient is 0.000, reaching an obvious level. The KMO coefficient is 0.929, which is greater than 0.900. The Bartlett spherical test shows that there are relatively more common factors between the 27 projects. It shows that the validity of the questionnaire is good, and that the data are suitable for factor analysis. As a result of the principal component analysis, six common factors with a cumulative contribution rate of 60.80% were extracted from the 27 questions in our online survey. The pattern and structures of the rotated common factors are shown in Table 2.

**Table 2:** Results of the factor analysis of 2058 medical students

Item	Common factors						Name offactors
	1	2	3	4	5	6	
I have no motivation for studying. I lack of perseverance.	0.752						Self-learning
I did not study very well, so I always make effort at the last moment and stay up all night for studying.	0.739						
When I am studying, I often fail to complete the task in the prescribed time.	0.734						
When I am studying in school, if the environment is a little noisy, I will be agitated and stop learning.	0.709						
The management style of college is not as good as middle school, and I feel less self-control.	0.651						
I find it difficult to relate real life to practical applications.	0.640						
I study very passively. I rarely ask questions or speak actively in class. I do not want to study actively.	0.632						
I often attend various learning activities and listen to some lectures about learning methods and experience.		0.776					Information utilization
I often ask other people how to fit the university’s study better.		0.755					
I often focus on the information inside and outside the school and try to increase the amount of information what I have.		0.679					
I can make friends from different majors and can learn and communicate with them.		0.671					
I can make good use of library’s books.		0.639					
I often learn from other people’s excellent learning experience to improve.		0.555					
I have a lot of own time in college, and I can use it very well.		0.447					
If I have a chance, I will go to a summer social practice.		0.423					
Although I feel nervous and anxious when I’m leaning, I can generally find ways to relaxing.			0.737				Environmental choice
I can adapt quickly to the new learning environment in university.			0.650				
I can adapt quickly to the new learning environment in university.			0.621				
I can use different learning methods according to the needs of university’ s study.			0.614				
Even if my learning environment is not good, I can create a good learning environment by myself.			0.562				
I think learning is important for the future work and life, so my self-consciousness is very high.				0.684			Goal orientation
I often develop a learning goal that combines long-term and short-term goals.				0.651			
I will make adjustments if my own learning goals are not realized.				0.573			
I feel that I’m under a lot of pressure, and I don’t know what to do to solve the problem.					0.868		Coping with stress
I’m worried about my career prospects, and I think there is a contradiction between the uncertain future job and the current hard work.					0.851		
I always want to change my major.						0.778	Professional interest
Because I am not interested in my professional courses, so my enthusiasm for learning has been affected.						0.737	

According to the original scale, the factors are identified as ‘self-learning,’ ‘information utilization,’ ‘environmental choice,’ ‘goal orientation,’ ‘coping with stress,’ and ‘professional interest.’ Self-learning refers to initiative, conscious and independent learning, and the intentional decision to manage learning autonomously. Information utilization refers to the effective use of information resources, such as libraries, the Internet, and human resources. Environmental choice refers to good decision-making about the learning environment, and developing effective learning behaviour. Goal orientation means that students have clear, attainable, and reasonable learning goals, which they can adjust if necessary. Coping with stress refers to the ability to manage educational, examination, and job pressure proactively and effectively. Professional interest refers to the enthusiasm and interest in the topic, and the tendency to seek knowledge.

### Correlations among study variables

Table 3 shows the means, standard deviations and Pearson correlation analysis of continuous variables. The results show that there is a significant correlation between the variables. The results indicate that medical students’ academic adaptability (Mean = 3.33), immersion in learning (Mean = 3.20) and academic performance (Mean = 3.39) were in the middle level, but academic burnout (Mean = 2.17) was at a low level. There is a good correlation between academic adaptability and academic burnout (*P*<0.01), immersion in learning (*P*<0.01) and academic performance (*P*<0.01).

**Table 3:** Means, standard deviation (SD) and correlations of continuous variables

	Mean	SD	1	2	3	4
1.Academic adaptability	3.33	0.46	1			
2.Academic burnout	2.17	0.81	−0.705^**^	1		
3.Immersion in learning	3.20	0.64	0.657^**^	−0.592^**^	1	
4.Academic performance	3.39	0.97	0.389^**^	−0.322^**^	0.306^**^	1

**P < 0.01 (two-tailed)

Table 4 shows that different demographic variables are tested using the *t* test or variance analysis. The results show that there are significant differences in gender (*t* = 2.857, *P* < 0.05), age (*F* = 1.883, *P* < 0.05), and grade (*F* = 3.878, *P* < 0.05).

**Table 4:** Differences in learning adaptability of medical students with different demographic variables

	*t/F*	*P*
Gender	2.857	0.000
Age	1.883	0.043
Grade	3.878	0.004
Student type	−5.399	0.274
Residence	1.270	0.690

Table 5 shows, using the hierarchical linear regression analysis method, that academic burnout, immersion in learning, and learning achievement were regarded as outcome variables, and learning adaptability as cause variables. Meanwhile, the gender, age, and grade of the sample were regarded as control variables in the regression model, aiming at eliminating the mixed effects (M1) and examining the effects of medical students’ academic adaptability on academic burnout, immersion in learning, and academic performance (M2). The results showed that the academic adaptability of medical students had a significant negative predictive effect on academic burnout (Beta = − 0.705, *P*<0.01). Academic adaptability had a significant predictive effect on immersion in learning (Beta = 0.655, *P*<0.01) and academic performance (Beta = 0.407, *P*<0.01).

**Table 5:** Regression model for academic adaptability’s impact on academic burnout, immersion in learning and academic performance

Variable	Academic Burnout		Immersion in Learning		Academic Performance	
Control variable	M1	M2	M1	M2	M1	M2
Gender	−0.003	0.045^**^	−0.013	0.026	0.179^**^	0.203^*^
Age	0.033	0.060^*^	0.053	0.028	0.079^*^	0.063
Grade	0.088^*^	0.010	−0.130^**^	−0.057^*^	−0.018	0.027
Causal variable						
Academic adaptability		−0.705^**^		0.655^**^		0.407^**^
*F*	8.945	502.355^**^	6.168	377.155^**^	23.098	121.658^**^
*R*^2^	0.013	0.505^**^	0.009	0.433^**^	0.034	0.198^**^
*△R*^2^	0.013	0.491^**^	0.009	0.424^**^	0.034	0.164^**^

Note: Academic burnout, immersion in learning, and academic performance are the control variables. Beta represents the standardized regression coefficient in the equation

^*^P<0.05 (two-tailed), ^**^P<0.01 (two-tailed)

## Discussion

The results show that there are significant differences in gender (*t* = 2.857, *P* < 0.05), age (*F* = 1.883, *P* < 0.05), and grade (*F* = 3.878, *P* < 0.05). There was no significant difference in student type (*t* = − 5.399, *P* > 0.05) and birth place (*t* = 1.270, *P* > 0.05).

The repetition of academic burnout, immersion in learning, and academic performance as significant factors associated with academic adaptability throughout the three regression models indicates the existence of a stable relationship among those factors.

From this study, we can see that there is a significant negative correlation between medical students’ academic learning adaptation and academic burnout (Beta = − 0.705, *P* < 0.01). There is a significant correlation between academic adaptability and academic burnout (r = − 0.705, *P* < 0.01). Therefore, academic burnout can be predicted through learning adjustment. Academic adaptation is a long-term process of self-adjustment, and academic burnout is also a changing psychological variable, so learning adaptation affects academic burnout inevitably. If students cannot adapt quickly to their studies and life, they will become incompatible, which can easily lead to learning disabilities and academic burnout. The better the academic adaptability, the less the likelihood of improper learning behaviour and depression. In the education process, the university’s intervention in medical students’ academic burnout should depend on individuals and should provide them with varying help and support. In addition, in order for individuals to avoid academic burnout, self-regulation, stimulation of learning motivation, a good attitude, and interest cultivation are effective ways for students to maintain a balance between learning activities and the learning environment. In conclusion, the better the academic adaptability of medical students is, the less likely they are to experience academic burnout. On the contrary, failure to adapt to studies and life at the university stage as soon as possible, results in maladjustment, which leads to learning difficulties and academic burnout.

In this study, academic adaptability had a significant positive correlation with immersion in learning (Beta = 0.655, *P*<0.01). The better the academic adaptability of medical students, the more their learning engagement and immersion in learning.

Academic adaptability can predict students’ academic performance (Beta = 0.407, *P* < 0.01) and has a significant correlation with academic performance (r = 0.389, *P* < 0.01). The better the academic adaptability of medical students, the greater their interest in learning and the higher their academic performance. Moreover, if students fail to adapt quickly to university life and study, they will not adjust well and will be more likely to perform poorly in their academics. According to previous studies, the adaptability of college students not only affects their psychological state and mental health but also their academic performance [6, 19]. Students with a high degree of academic adaptability can always achieve good results not only in examinations but also in areas such as obtaining the approval of teachers and parents, in the pursuit of their goals, and in obtaining scholarships. These results increase academic adaptability which could grow their confidence imperceptibly and increase their self-expectations. Students with low grades have a low independent learning ability, low motivation to learn, and a low self-perception, which leads some medical students to lower levels of academic adaptability creating a vicious cycle. However, because of the cross-sectional study, it is impossible to determine the causal relationship between academic adaptability and academic performance. In other words, the higher the academic adaptability, the better the students’ academic performance. It can also be seen from the side that the validity of this scale is good.

This study had some limitations. First of all, the sample selection of this study has limitations. It uses a combination of stratified sampling and convenient sampling to conduct a partial survey of Harbin Medical University, Jiamusi University College of Medicine, Qiqihar Medical College, and Chengdu Medical College. A balanced survey of all grades will inevitably lead to uneven sampling, which will have a certain impact on data quality. In terms of data collection, this study publishes and recycles questionnaires through the ‘Questionnaire Star’ network platform. Answering questions on-site, doubts, and inability to distribute and collect questionnaires make it impossible to grasp or supervise the answering environment of the respondents and whether the respondents independently answered the questions. There was a problem with the quality of some of the questionnaires recovered; and this study is limited by the research objective conditions of the research sampling institute The medical schools are located in Heilongjiang Province. There is only one key medical school, that is, Harbin Medical University. Because of the quality of students and geographical factors, the application of the conclusions of the research may be limited, and the response may be affected by personal bias. In terms of results analysis, because the cross-sectional nature of this study cannot effectively and accurately judge the causal relationship, the conclusions of the results are limited. Responses might have been influenced by personal bias. In addition, because of the limitation of individual ability, the pre-study understanding of the literature on academic adaptability is mastered. It is not comprehensive enough, and the data collection and data processing are not deep enough. Additionally, the results suggestion part has yet to be further improved.

## Conclusion

This survey showed that medical students’ academic adaptability had a significant effect on academic burnout, immersion in learning, and academic performance. Medical students who have good academic adaptability will encounter less education burnout, more immersion in learning, and better academic performance. Looking at academic adaptability and ways to enhance such skills might be helpful for medical schools to enhance student performance and engagement while in school.

## Data Availability

The datasets used and/or analyzed during the current study are available from the corresponding author on reasonable request.

## Abbreviations

MSAA: Medical Students’ Academic Adaptability
SPSS: Statistical Package for Social Sciences
